# Effectiveness of an educational program on improving the knowledge and practice of environmental sustainability in dentistry among undergraduate students at Faculty of Dentistry in Egypt: an interventional study

**DOI:** 10.1186/s12909-025-08137-z

**Published:** 2026-01-05

**Authors:** Esraa Mahmoud Bishr, Mohamed Fakhry Hussein, Gaber Abuzaid Ismail, Ebtisam Mohamed Fetohy, Aleya Hanafy El-Zoka

**Affiliations:** 1 Department of Occupational Health and Industrial Medicine, High Institute of Public Health, Alexandria University, Alexandria, Egypt; 2 Department of Dental Public Health and Preventive Dentistry, Faculty of Dentistry, Pharos University, Alexandria, Egypt; 3 Department of Environmental Health, High Institute of Public Health, Alexandria University, Alexandria, Egypt; 4 Department of Health Administration and Behavioral Sciences, High Institute of Public Health, Alexandria University, Alexandria, Egypt

**Keywords:** Sustainable dentistry, Eco-dentistry, Sustainable development, Educational program, Knowledge, Practice, Egypt

## Abstract

**Background:**

Dental practices generate significant environmental impacts, necessitating environmentally sustainable dentistry (ESD) for equitable, ethical, and resource-efficient care. However, a knowledge gap exists among dental practitioners regarding sustainable practices.

**Objectives:**

This study assessed the effectiveness of an educational program on the knowledge and practice (KP) of undergraduate dental students at Pharos University in Alexandria (PUA), Egypt, regarding ESD.

**Methods:**

The study was conducted from April to November 2024 and recruited 175 final pre-clinical (3rd) and final clinical (5th) year students. Participants' KP was evaluated before, immediately after, and six months post-program using a structured questionnaire. Statistical analysis employed t-tests, one-way ANOVA, and univariate regression models.

**Results:**

Participants (58.28% female, mean age 20.88 years) initially demonstrated low awareness of sustainability, primarily relying on social media for information (37.14%). The program improved knowledge, increasing the mean percentage from (60.38% ± 17.74%) to (92.60% ± 7.42%) immediately post-program and (76.41% ± 21.66%) after six months (*p* < 0.001). Self-reported practices also improved, rising from (52.84% ± 10.79%) to (64.14% ± 11.73%) then (61.90% ± 13.47%) (*p* < 0.001). Participants with good KP increased from (12.57%, 1.14%) pre-program to (93.14%, 15.42%) immediately post-program, then (51.42%, 18.28%) after six months (*p* < 0.001). Moderate positive correlations were found between knowledge and practice post-intervention (*p* < 0.05). Regression indicated that prior awareness of sustainability strongly predicted better KP of ESD at baseline, also final clinical educational level predicted good knowledge and using social media/websites predicted good practice.

**Conclusions:**

Baseline KP regarding ESD was low. The educational program significantly enhanced participants' KP, demonstrating the potential of the multi-component targeted educational intervention in enhancing ESD. Incorporating sustainability into undergraduate curricula is recommended to improve future dental professionals' competencies.

**Trial registration:**

The clinical trial registration number is PACTR202406623852822 (Date: 12 June 2024), “retrospectively registered”.

**Supplementary Information:**

The online version contains supplementary material available at 10.1186/s12909-025-08137-z.

## Background

The oral health care (OHC) field, with its significant resource consumption and environmental impact, is a notable contributor to climate change and other global challenges [1, 2]. Addressing this requires a holistic strategy that extends beyond conventional measures. A truly sustainable future demands a "triple bottom line" approach, which integrates environmental, social, and economic responsibility [3]. This framework aligns directly with the United Nations' Sustainable Development Goals (SDGs), a blueprint of 17 goals aimed at creating a more equitable and sustainable future by 2030 [4]. To achieve these goals, dental practitioners must become active participants in sustainable OHC. The World Dental Federation (FDI) has defined this as the provision of equitable, ethical, and high-quality care that ensures the efficient use of resources while minimizing environmental impact. This new framework, now widely known as Environmentally Sustainable Dentistry (ESD), represents a fundamental and necessary shift in modern dental practice [1, 2, 5].

To understand why this shift is necessary, it is crucial to examine the dental profession’s environmental footprint. Dentistry, while playing a crucial role in improving community oral health and preventing diseases, consumes large amounts of resources in the form of energy, water, and single-use items. This, along with the generation of solid and hazardous waste, plus travel to and from facilities contribute to its environmental footprint [5]. For instance, NHS dental services in England generate an estimated 675,706 tonnes of carbon dioxide equivalent (tCO_2_eq) annually, which is comparable to 50,000 flights from the UK to Hong Kong [1, 6]. Similarly, a study in Alexandria and Elbeheira, Egypt, revealed an average annual carbon footprint of 14,426.8 kg CO_2_eq per private clinic and 4.3 kg CO_2_eq per patient visit [7]. Despite positive measures like technological advancements, and waste segregation regulations, many routine procedures and materials still carry a significant environmental burden [5].

To address these challenges, dentists are ethically and professionally obligated to adopt ESD, guided by the 4Rs approach: Rethink, Reduce, Reuse, and Recycle [2]. This framework involves rethinking, which encourages dental professionals to critically evaluate existing practices and consider innovative, eco-friendly alternatives to traditional methods. This approach also includes reducing resource use by utilizing tele-dentistry, digitalization, technological devices, energy-saving devices, and dry dental vacuum pumps, as well as decreasing single-use items by switching to autoclavable alternatives. A circular economy is embraced through recycling materials like base-metal casting alloys, waxes, gypsum, and alginate. Moreover, placing the highest priority on preventing oral diseases and promoting oral health has positive impacts on both patients and the environment. This comprehensive approach, supported by external drivers like policy, research, and education, is essential for achieving sustainable dental practice [1, 2, 5, 8].

While solutions and frameworks for ESD exist, their widespread adoption is hindered by significant gaps in professional awareness. This barrier has been identified as the greatest challenge to ESD and the most significant opportunity for impactful change [8, 9]. Professional bodies like the FDI and Association for Dental Education in Europe (ADEE) have formally recommended integrating sustainability and green practices into dental curricula [2, 10, 11], a call supported by cross-sectional studies that report low knowledge but high willingness to learn among students, practitioners, and educators in the US & UK [12–14], India [15], UAE [16, 17], KSA [18], and Egypt [19]. Although successful educational interventions have been conducted in places like the UK [20, 21], USA [22], KSA [23], and Peru [24], a comprehensive review by Bamedhaf et al. [25] highlights that the number of such studies remains limited, which has also been noted in Egypt. This underscores the need for more targeted research and educational initiatives to bridge this persistent knowledge gap.

Therefore, this study aims to evaluate the effectiveness of a targeted educational program on KP of undergraduate dental students at a dental faculty in Alexandria, Egypt, concerning ESD. It is hypothesized that this program will significantly improve the KP levels of the study group.

## Methods

### Aim of the study

The current study aimed to design, implement and assess effectiveness of an educational program intended to improve the KP of undergraduate students at Faculty of Dentistry, PUA regarding ESD, using a comprehensive questionnaire to evaluate both the understanding and implementation of these principles at baseline, immediately after program implementation then after six months to evaluate program effectiveness overtime.

### Study design and setting

An interventional study, single group (pre-test/post-test) design, took place between April and November 2024, at Faculty of Dentistry, PUA, Egypt.

### Target population

The target population was undergraduate dental students enrolled in the final preclinical (3rd) and final clinical (5th) years. These two key cohorts were selected because they represent students who have collective knowledge and skills from the dental educational program and so possess the requisite practical knowledge for a comprehensive assessment. Exclusion criteria included freshmen, sophomore and mid-senior students (1st, 2nd and 4th years) and those on academic probation or suspension. Participation was voluntary.

### Sample size and method of selection

The sample size was calculated using OpenEpi (version 3.0), an open-source sample size calculator, with a 95% confidence level, 5% alpha error, and an expected proportion of 87.31% for correct knowledge regarding the use of amalgam vacuum filters [26]. The sample size was estimated to be 146, considering a 20% drop-out ratio; the sample size increased to 175 students.

Participants were recruited through a systematic random sampling technique from a complete list of 350 eligible students. A random starting point was selected, and every second student was chosen from the list until the desired sample size of 175 was reached. This method ensured a representative sample from both student cohorts, with 142 third-year students and 33 fifth-year students being included. Students were then divided into 6 groups (~ 30 students each) for the intervention program.

### Data collection tools (Supplementary material I)

The pre-designed, structured, self-administered questionnaire was constructed by the researchers, based on the clinical guidelines of FDI for sustainable dentistry and guidelines for environmental sustainability [2, 27], to assess KP regarding ESD. The content validity was then established by a panel of expert professors in public health, dentistry and health education.

### Pilot feasibility and reliability study

A preliminary study was conducted with 17 dental students who met the same eligibility criteria as the main study participants but were excluded from the final sample. The purpose of this study was to assess the clarity and feasibility of the questionnaire and to estimate the average completion time at approximately 10 min. The tool's acceptable internal reliability was confirmed with a Cronbach’s alpha of 0.71.

The questionnaire consisted of three sections:

- The first section collected socio-demographic data (7 questions)—Participants also provided a self-assessment of their awareness of sustainable dentistry, indicating any previous involvement in related activities or workshops and their sources of information.
- The second section assessed the participants’ knowledge about ESD (18 multiple-choice questions). It included questions about 4Rs, green dentistry, the application of these concepts regarding use of dental materials and equipment, SWM, hazardous waste, and role of preventive dentistry in sustainability. Each question was scored 0 (incorrect/don't know) or 1 (correct). Total knowledge scores ranged from 0 to 18. Knowledge levels were categorized using Bloom's cut-off points: good (15–18, 80–100%), moderate (11–14, 60–79%), and poor (< 11, < 60%) [28].
- The third section evaluated self-reported practices related to ESD (22 questions). It inquired about the frequency of specific behaviors, including transportation methods, energy and water conservation, purchasing habits, disposable instrument usage, waste separation, hazardous material management, and patient health education. Responses were scored as follows: 2 for 'always,' 1 for 'sometimes,' and 0 for 'never.' Total practice scores ranged from 0 to 44. Practice levels were then categorized: good (> 33 points, > 75%), fair (22–33 points, 50–75%), and poor (< 22 points, < 50%). To ensure consistency and accurate interpretation, all negatively worded or reversed questions within the questionnaire were appropriately re-coded prior to statistical analysis.

### Program structure and intervention phase

After identifying key areas of KP gaps through the initial questionnaire, a targeted educational program was developed and delivered by researchers. The program consisted of three sessions (one-hour each), delivered over three consecutive weeks, immediately after class hours, at the lecture hall in the main building of the Faculty of Dentistry, PUA.

The intervention was structured as a multi-component educational program, which incorporated a variety of interactive teaching methods to promote comprehensive learning. Each session used a mix of these methods—including PowerPoint presentations, videos, brainstorming, group discussions, real-life scenarios, games and role-playing—to reinforce learning.

Session 1 introduced the foundations of sustainable OHC. It covered the SDGs, the impact of climate change on OHC, and the "4Rs" through interactive presentations and participatory learning activities. Session 2 focused on carbon footprint of OHC and sustainable practices, including the management of hazardous materials and tools. This session incorporated competitive games, interactive lectures, and audiovisual aids. The final session explored innovations and future directions in sustainable dentistry, such as recycling, digital technologies, and preventive dentistry. It was primarily driven by open discussions with visual aids.

To evaluate the program's effectiveness, the paper-based pre-intervention questionnaire was re-administered immediately after the final session. Then for the follow-up, after six months, the same questionnaire was converted to an online Google Form. The researcher met with students on campus and provided a QR code that they scanned to complete the survey on their mobile devices on spot. This eco-friendly approach eliminated paper waste and facilitated more efficient and accurate data collection by removing the need for manual data-entry.

### Ethical considerations

The research was approved by the Ethics Committee of High Institute of Public Health, Alexandria University (IRB: 00013692, Serial No.: AU09244302124, Date: 30/4/2024). It followed the International Guidelines for Research Ethics outlined in the 1964 Declaration of Helsinki and its subsequent amendments. The clinical trial registration number is PACTR202406623852822 (Date: 12 June 2024), “retrospectively registered”. All participants provided written informed consent after being informed of the study purpose and their right to withdraw. Anonymity and confidentiality were maintained throughout.

### Statistical analysis

The statistical analysis of the data was performed using IBM SPSS software version 20.0 (Armonk, NY: IBM Corp, released 2011). Categorical data were summarized as numbers and percentages. For continuous data, normality was assessed using the Kolmogorov–Smirnov test. Quantitative data were described using range (minimum and maximum), mean and standard deviation (SD). The significance of the results obtained was judged at the 5% level. Statistical tests employed were Chi-square test for categorical variables, to compare between different groups, Student t-test for normally distributed quantitative variables, to compare between two studied groups, ANOVA with repeated measures for normally distributed quantitative variables, to compare between more than two periods, and Friedman test to compare between more than two periods or stages. Normality of the data was confirmed with the Shapiro–Wilk test and checked for outliers using scatterplots and box plots. The data were normally distributed with no significant outliers; this justifies the use of Pearson’s correlation to assess the relationship between knowledge and practice. Univariate logistic regression models were implemented to identify factors affecting good KP.

## Results

Table 1 presents the socio-demographic characteristics of the 175 undergraduate dental students. Ages ranged from 19–23 years, with a mean age of 20.88 ± 1.16 years. Females comprised 102 (58.28%) of the sample with 142 (81.11%) residing in urban areas. The majority (142, 81.14%) were in their third (final pre-clinical) year, while 33 (18.86%) were in their fifth (final clinical) year.

**Table 1:** Distribution of participants according to sociodemographic characteristics and awareness about sustainability, (*n* = 175)

Sociodemographic characteristics & awareness about sustainability	No N = 175	%
Age		
≤ 20	87	49.71
> 20	88	50.28
Min – Max	19–23	
Mean ± SD	20.88 ± 1.16	
Educational Level		
Pre-clinical	142	81.14
Clinical	33	18.86
Sex		
Male	73	41.71
Female	102	58.28
Area of residence		
Urban	142	81.11
Rural	33	18.85
Previous participation in events or activities about “Sustainable Development”		
No	138	78.85
Yes	37	21.14
Awareness about the concept of 'sustainability' in dentistry		
Never heard about the concept of sustainability	73	41.71
Only heard about the concept of sustainability	60	34.28
Have some knowledge about the basic concept	40	22.85
Received training or have good experience with the sustainability approach and can teach it to others	2	1.14
Source of knowledge about ‘Sustainability’ in dentistry		
Social-Media & Websites	65	37.14
No source	44	25.14
From this questionnaire	38	21.71
University curriculum	17	9.71
Awareness campaigns & trainings	9	5.14
Colleagues & relatives	6	3.42
Other	4	2.28

Regarding sustainability awareness, 138 (78.85%) of participants reported no prior engagement in related activities. A large proportion of participants either never heard about the concept or only had basic understanding about sustainability in dentistry (73 or 41.71% and 60 or 34.28%, respectively). Less than a quarter of the participants (40 or 22.85%) possessed some information about it. Regarding the sources that participants acquired their sustainability knowledge through; social media was the primary information source (65 or 37.14%), followed by the study questionnaire itself (38 or 21.71%). Notably, 44 (25.14%) of participants were unable to identify any information source.

Table 2 presents the distribution of participants’ mean scores for each knowledge question and the overall knowledge scores across the three study phases.

**Table 2:** Distribution of participants’ mean scores of each knowledge question and the overall knowledge scores across the three study phases, (18Q and total), (*n* = 175)

Knowledge	Pre-intervention	Immediate post-intervention	Six months post-intervention	F	P	p_1_	p_2_	p_3_
	Mean ± SD	Mean ± SD	Mean ± SD					
1 (Solid waste & 4R)	0.90 ± 0.30	0.99 ± 0.11	0.94 ± 0.23	7.096^*^	0.002^*^	< 0.001^*^	0.351	0.061
2 (Waste segregation)	0.78 ± 0.42	0.95 ± 0.21	0.83 ± 0.37	12.719^*^	< 0.001^*^	< 0.001^*^	0.499	< 0.001^*^
3 (Waste disposal & pollution)	0.92 ± 0.27	0.99 ± 0.11	0.94 ± 0.23	4.767^*^	0.017^*^	0.007^*^	1.000	0.013^*^
4 (Limited water resources)	0.57 ± 0.50	0.97 ± 0.17	0.83 ± 0.38	71.956^*^	< 0.001^*^	< 0.001^*^	< 0.001^*^	< 0.001^*^
5 (Energy & climate change)	0.90 ± 0.30	0.99 ± 0.11	0.92 ± 0.27	6.731^*^	0.002^*^	0.001^*^	1.000	0.004^*^
6 (Impact of energy consumption)	0.42 ± 0.49	0.79 ± 0.41	0.62 ± 0.49	34.445^*^	< 0.001^*^	< 0.001^*^	< 0.001^*^	0.002^*^
7 (Climate change & oral health)	0.81 ± 0.39	0.99 ± 0.11	0.94 ± 0.24	20.705^*^	< 0.001^*^	< 0.001^*^	0.001^*^	0.036^*^
8 (Carbon footprint)	0.47 ± 0.50	0.98 ± 0.15	0.83 ± 0.38	100.016^*^	< 0.001^*^	< 0.001^*^	< 0.001^*^	< 0.001^*^
9 (Sustainability)	0.45 ± 0.50	0.90 ± 0.30	0.74 ± 0.44	61.751^*^	< 0.001^*^	< 0.001^*^	< 0.001^*^	< 0.001^*^
10 (Circular economy)	0.34 ± 0.47	0.70 ± 0.46	0.56 ± 0.50	30.877^*^	< 0.001^*^	< 0.001^*^	< 0.001^*^	0.017^*^
11 (Green dentistry)	0.51 ± 0.50	0.95 ± 0.24	0.70 ± 0.46	58.836^*^	< 0.001^*^	< 0.001^*^	< 0.001^*^	< 0.001^*^
12 (Carbon footprint in dentistry)	0.45 ± 0.50	0.97 ± 0.18	0.65 ± 0.48	73.341^*^	< 0.001^*^	< 0.001^*^	< 0.001^*^	< 0.001^*^
13 (Dental materials)	0.63 ± 0.48	0.90 ± 0.30	0.76 ± 0.43	22.406^*^	< 0.001^*^	< 0.001^*^	0.008^*^	0.002^*^
14 (Sustainable dental practice)	0.49 ± 0.50	0.95 ± 0.21	0.73 ± 0.44	64.518^*^	< 0.001^*^	< 0.001^*^	< 0.001^*^	< 0.001^*^
15 (Hazardous dental waste)	0.74 ± 0.44	0.99 ± 0.08	0.79 ± 0.41	31.158^*^	< 0.001^*^	< 0.001^*^	0.570	< 0.001^*^
16 (X-ray films)	0.37 ± 0.48	0.75 ± 0.44	0.54 ± 0.50	35.035^*^	< 0.001^*^	< 0.001^*^	0.001^*^	< 0.001^*^
17 (Amalgam waste)	0.45 ± 0.50	0.95 ± 0.22	0.65 ± 0.48	77.710^*^	< 0.001^*^	< 0.001^*^	< 0.001^*^	< 0.001^*^
18 (Intra-oral scanners)	0.69 ± 0.46	0.97 ± 0.18	0.78 ± 0.41	28.850^*^	< 0.001^*^	< 0.001^*^	0.096	< 0.001^*^
Total score (0–18)								
Min. – Max	1–18	12–18	0–18	213.363*	< 0.001*	0.001*	0.001*	0.285
Mean ± SD	10.87 ± 3.19	16.67 ± 1.34	13.75 ± 3.90					
Percentage score (0–100%)								
Min. – Max	5.56–100.0	66.67–100.0	0.0–100.0					
Mean ± SD	60.38 ± 17.74	92.60 ± 7.42	76.41 ± 21.66					

*SD* Standard deviation

Fr: Friedman test, Significance between periods were done using Post Hoc Test (Dunn's)

p_1_: *p* value for comparing between pre-intervention and immediate post-intervention

p_2_: *p* value for comparing between pre-intervention and six months post-intervention

p_3_: *p* value for comparing between immediate post-intervention and six months post-intervention

p: *p* value for comparing between the studied periods

^*^Statistically significant at *p* < 0.05

### Overall knowledge analysis

The overall mean knowledge score showed a significant improvement from baseline (10.87 points, 60.38%) to the immediate post-test (16.67 points, 92.60%). This improvement remained statistically significant at the 6-month follow-up (13.75 points, 76.41%) when compared to the baseline score (*p* < 0.001).

### Individual question analysis

Baseline scores reveal a dual level of knowledge. Participants demonstrated a strong foundational understanding of traditional topics, as evidenced by high mean scores on questions related to conventional waste management (e.g., Q1: 0.90 ± 0.30; Q3: 0.92 ± 0.27). In contrast, questions related to newer sustainability concepts had lower baseline scores. Following the intervention, a statistically significant increase (*p* < 0.005) was observed for all 18 questions between the pre-test and immediate post-test. Although a slight decline in mean scores occurred at the 6-month follow-up, 12 questions (Q4, Q6-Q14, Q16, Q17) still showed significantly higher scores than baseline (*p* < 0.005).

Table 3 shows the distribution of participants’ mean scores of each self-reported practice and the overall practice scores across the three study phases.

**Table 3:** Distribution of participants’ mean scores for each self-reported practice and the total practice mean scores, across the three study phases, (22Q and total), (*n* = 175)

Practice	Pre-intervention	Immediate post-intervention	Six months post-intervention	F	*p*	p_1_	p_2_	p_3_
	Mean ± SD	Mean ± SD	Mean ± SD					
Q1 (means of transportation)	0.43 ± 0.61	0.50 ± 0.53	0.67 ± 0.63	12.708^*^	< 0.001^*^	0.541	< 0.001^*^	0.002^*^
Q2 (active transportation)	1.05 ± 0.58	1.16 ± 0.52	1.16 ± 0.57	3.692^*^	0.026^*^	0.045^*^	0.069	1.000
Q3 (energy conservation)	1.60 ± 0.65	1.71 ± 0.50	1.61 ± 0.59	2.479	0.085	–	–	–
Q4 (clean energy)	0.65 ± 0.71	0.82 ± 0.67	0.97 ± 0.70	11.740^*^	< 0.001^*^	0.026^*^	< 0.001^*^	0.097
Q5 (water conservation)	0.78 ± 0.73	1.17 ± 0.66	1.13 ± 0.69	22.493^*^	< 0.001^*^	< 0.001^*^	< 0.001^*^	1.000
Q6 (paper printing)	1.09 ± 0.69	1.39 ± 0.62	1.31 ± 0.67	13.798^*^	< 0.001^*^	< 0.001^*^	0.001^*^	0.595
Q7 (paper consumption)	1.19 ± 0.74	1.42 ± 0.66	1.35 ± 0.70	6.839^*^	0.001^*^	0.001^*^	0.065	0.642
Q8 (purchasing)	0.91 ± 0.69	1.21 ± 0.69	1.12 ± 0.70	12.110^*^	< 0.001^*^	< 0.001^*^	0.002^*^	0.488
Q9 (eco-friendly manufacture)	1.14 ± 0.65	1.33 ± 0.64	1.24 ± 0.66	5.603^*^	0.004^*^	0.003^*^	0.233	0.361
Q10 (recycled products)	0.99 ± 0.57	1.15 ± 0.59	1.09 ± 0.58	4.373^*^	0.013^*^	0.011^*^	0.232	0.694
Q11 (disposable instruments)	1.03 ± 0.63	1.18 ± 0.60	1.25 ± 0.64	6.575^*^	0.002^*^	0.046^*^	0.003^*^	0.825
Q12 (disposable pouches)	0.36 ± 0.58	0.52 ± 0.63	0.66 ± 0.72	11.157^*^	< 0.001^*^	0.037^*^	< 0.001^*^	0.089
Q13 (sterilizable tools)	1.13 ± 0.70	1.30 ± 0.69	1.15 ± 0.70	3.333^*^	0.037^*^	0.017^*^	0.822	0.033^*^
Q14 (waste segregation)	1.35 ± 0.73	1.64 ± 0.64	1.51 ± 0.68	11.067^*^	< 0.001^*^	< 0.001^*^	0.045^*^	0.099
Q15 (sharp waste)	1.62 ± 0.63	1.73 ± 0.55	1.66 ± 0.57	2.251	0.107	–	–	–
Q16 (x-ray film)	0.77 ± 0.78	1.27 ± 0.78	1.10 ± 0.75	23.811^*^	< 0.001^*^	< 0.001^*^	< 0.001^*^	0.044^*^
Q17 (capsulated amalgam)	1.40 ± 0.75	1.47 ± 0.63	1.54 ± 0.61	2.490	0.084	–	–	–
Q18 (amalgam procedures)	1.10 ± 0.81	1.34 ± 0.72	1.37 ± 0.65	9.439^*^	< 0.001^*^	0.001^*^	0.001^*^	1.000
Q19 (amalgam solid waste)	1.05 ± 0.81	1.47 ± 0.73	1.29 ± 0.75	17.287^*^	< 0.001^*^	< 0.001^*^	0.008^*^	0.008^*^
Q20 (amalgam waste water)	1.26 ± 0.78	1.48 ± 0.72	1.42 ± 0.70	5.425^*^	0.005^*^	0.009^*^	0.058	1.000
Q21 (x-ray fixer waste water)	1.09 ± 0.82	1.39 ± 0.80	1.25 ± 0.77	8.335^*^	< 0.001^*^	< 0.001^*^	0.079	0.782
Q22 (preventive dentistry)	1.27 ± 0.65	1.57 ± 0.55	1.39 ± 0.66	11.961^*^	< 0.001^*^	< 0.001^*^	0.210	0.014^*^
Total score (0–44)								
Min. – Max	2–34	15–41	13–40	58.350*	< 0.001*	< 0.001*	< 0.001*	0.076
Mean ± SD	23.25 ± 4.75	28.22 ± 5.16	27.23 ± 5.93					
% Score								
Min. – Max	4.55–77.27	34.09–93.18	29.55–90.91					
Mean ± SD	52.84 ± 10.79	64.14 ± 11.73	61.90 ± 13.47					

*SD* Standard deviation

Fr: Friedman test, Significance between periods were done using Post Hoc Test (Dunn's)

p_1_: *p* value for comparing between pre-intervention and immediate post-intervention

p_2_: *p* value for comparing between pre-intervention and six months post-intervention

p_3_: *p* value for comparing between immediate post-intervention and six months post-intervention

p: *p* value for comparing between the studied periods

^*^Statistically significant at *p* < 0.05

Overall self-reported practice significantly improved post-program (*p* < 0.001). Total self-reported practice scores (range 0–44) increased from a baseline mean of 23.25 ± 4.75 (52.84% ± 10.79%) to 28.22 ± 5.16 (64.14% ± 11.73%) immediately post-intervention. While, at the 6-month follow-up, scores slightly decreased to 27.23 ± 5.93 (61.90% ± 13.47%) but remained significantly higher than baseline (*p* < 0.001) and no significant difference was found between immediate post-intervention and 6-month follow-up of total practice scores.

### Singular self-reported practice

Comparative analysis revealed significant improvements (*p* < 0.005) between pre-test and immediate post-test for 19 items; but no significant differences were observed for Q3, Q15, and Q17. When comparing pre-test and 6-month follow-up, significant differences persisted for 11 items [Q1, Q4, Q5, Q6, Q8, Q11, Q12, Q14, Q16, Q18, Q19]. Notably, six items [Q1, Q4, Q11, Q12, Q17, Q18] demonstrated higher mean scores at the 6-month follow-up than immediately post-intervention.

**Fig. 1:**
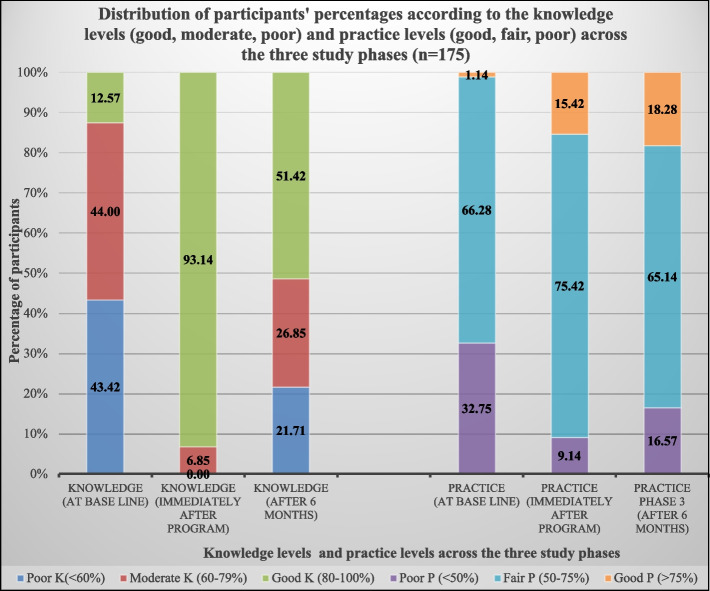
Distribution of participants’ percentages according to the total knowledge levels (good, moderate, poor) and total practice levels (good, fair, poor), across the three study phases (*n* = 175)

Figure 1 illustrates the changes in participants’ frequency and percentage according to the total knowledge levels (good, moderate, poor) and total practice levels (good, fair, poor) across the three study phases.

Initially, only 22 participants (12.57%) demonstrated good knowledge, while 77 (44.00%) had moderate knowledge and 76 (43.42%) had poor knowledge. Baseline self-reported practice levels were similarly low, with just 2 (1.14%) reporting good practices, 116 (66.28%) fair practices, and 57 (32.75%) poor practices. After the intervention, a marked improvement occurred, where 163 (93.14%) demonstrated good knowledge, 12 (6.85%) moderate knowledge, and no one (0.00%) demonstrated poor knowledge. Practice levels also improved, with 27 (15.42%) reporting good practices, 132 (75.42%) fair practices, and 16 (9.14%) poor practices. At the 6-month follow-up, while KP levels decreased slightly compared to immediately post-intervention, they remained significantly higher than baseline (*p* < 0.001). Specifically, 90 (51.42%) demonstrated good knowledge, and 32 (18.28%) showed good practice. A significant difference was observed between baseline and the 6-month follow-up (*p* < 0.001), but not between the post-intervention and 6-month follow-up."

**Fig. 2:**
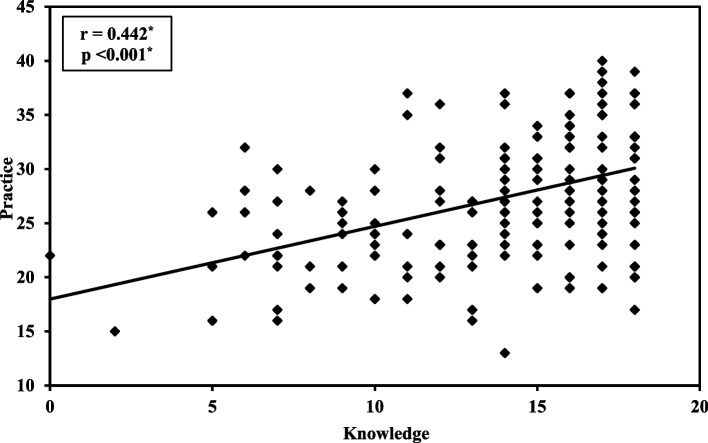
Correlation between knowledge and practice after 6 months of intervention, (*n* = 175)

Figure 2 demonstrates the relationship between total knowledge and practice scores, six-month post-intervention. A statistically significant moderate positive correlation was found (*r* = 0.442, *p* < 0.001), indicating that increased knowledge was correlated with increased practices scorers. For other correlations please refer to supplementary material II.

Table 4 presents the univariate logistic regression analysis, revealing key predictors of good KP. Participants in the final clinical (5th year) educational level were 2.872 times more likely to have good knowledge than younger participants in final preclinical (3rd year), (CI = 1.213–6.797, *p* < 0.05). Likewise, those possessing basic awareness of the concept of sustainability were 6.387 times more likely to exhibit good knowledge than those who never heard of the concept (CI = 2.499–16.324, *p* < 0.001). For good practice, participants who have heard about sustainability were 2.425 times more likely to perform good practice than those who have never heard of the concept (CI = 1.137–5.171, *p* < 0.05). Similarly, participants that used social media/websites as a source of information were 2.338 more likely to perform good practice (CI = 1.156–4.730, *p* < 0.05) compared to those who did not use this source.

**Table 4:** Univariate logistic regression analysis for the parameters affecting good knowledge and good practice, (*n* = 175)

Parameters affecting good knowledge and good practice	Univariate logistic regression	
	p	OR (LL – UL 95%C.I)
Good Knowledge (good & moderate) (no. = 76 vs. 99)		
Age	0.437	1.110 (0.853–1.443)
Educational Level [5th grade]	0.016^*^	2.872 (1.213–6.797)
Sex[female]	0.828	0.935 (0.510–1.715)
Area of Residency [Rural]	0.072	0.494 (0.229–1.065)
Participated in workshops	0.275	0.667 (0.322–1.381)
How do you assess 'sustainability'?		
I never heard about the concept of sustainability®		1.000
I only heard about the concept of sustainability	0.104	1.772 (0.889–3.532)
I have some knowledge about the basic concept	< 0.001^*^	6.387 (2.499–16.324)
I received training and have some experience, and I have good experience with the sustainability approach that I can teach it to others	0.832	1.355 (0.082–22.513)
Source of information		
University studies (curriculum)	0.478	1.458 (0.514–4.139)
Awareness campaigns/Trainings	0.205	2.815 (0.568–13.958)
Social media/Websites	0.482	1.250 (0.671–2.329)
Colleagues and relatives	0.211	3.989 (0.456–34.883)
From this questionnaire	0.854	0.934 (0.453–1.926)
Other	0.465	2.344 (0.239–22.988)
Good practice (good & fair) (no. = 118 vs. 57)		
Age	0.797	0.965 (0.735–1.267)
Educational Level [5th grade]	0.355	0.691 (0.315–1.512)
Sex[female]	0.089	1.742 (0.919–3.302)
Area of Residency [Rural]	0.758	1.138 (0.501–2.585)
Participated in workshops	0.231	1.659 (0.724–3.800)
How do you assess 'sustainability'?		
I never heard about the concept of sustainability®		1.000
I only heard about the concept of sustainability	0.022^*^	2.425 (1.137–5.171)
I have some knowledge about the basic concept	0.118	1.946 (0.844–4.484)
I received training and have some experience, and I have good experience with the sustainability approach that I can teach it to others	0.832	0.738 (0.044–12.264)
Source of information		
University studies (curriculum)	0.186	0.506 (0.184–1.389)
Awareness campaigns/Trainings	0.501	1.734 (0.349–8.627)
Social media/Websites	0.018^*^	2.338 (1.156–4.730)
Colleagues and relatives	0.413	2.478 (0.283–21.719)
From this questionnaire	0.590	1.241 (0.566–2.722)
Other	0.745	1.461 (0.149–14.363)

*OR* Odds ratio, *C.I* Confidence interval, *LL* Lower limit, *UL* Upper Limit

^#^All variables with *p* < 0.05

®Reference value

^*^Statistically significant at *p* < 0.05

## Discussion

The limited familiarity with environmental sustainability within the dental field, potentially stemming from a lack of awareness regarding dentistry's environmental impact, provoked this study to evaluate the effectiveness of an educational intervention on the knowledge and self-reported practices (KP) of 175 undergraduate dental students.

The study-sample demographics were compared to the broader student population at the Faculty of Dentistry in terms of gender ratio and age distribution, with a slightly higher percentage of females. The higher percentage of final preclinical 3rd year students to final clinical 5th year students, is a direct reflection of the actual student population sizes at the time of recruitment which ensured a representative sample of the study population.

At baseline, a significant deficit in sustainability expertise was obvious, with most students reporting no prior engagement, unfamiliarity with sustainability in dentistry, or only displaying basic awareness. Social media served as their primary information source, which, despite its predictive value for good practice, raises concerns about the reliability of the information received [29], while the questionnaire itself was the second most common information source, highlighting an existing knowledge gap. The limited knowledge derived from university studies, campaigns, colleagues, and other avenues indicates underutilization of these channels. While a small number of students (*n* = 17) cited university curricula as a source of information, this does not indicate the presence of a dedicated sustainable dentistry program. Their responses likely stem from indirect exposure to related concepts within existing subjects, such as protocols for waste disposal in infection control or the importance of preventive dentistry. The minimal number of students citing this source further reinforces that this information is fragmented rather than being part of a comprehensive, structured curriculum. Alarmingly, a notable proportion of participants could not identify any information source. This low baseline awareness aligns with findings among dental practitioners in Alexandria [19], US dental schools [12] and Saudi Arabian dental students and faculty, contrasting with higher awareness levels observed in Turkey [30] and among some Romanian practitioners [31]. Variations in the recognition of terms like 'green dentistry' and 'eco-dentistry' further underscore regional differences [15, 32–36]. Moreover, a review encompassing thirteen studies suggested good knowledge but inadequate implementation of sustainable dental practice globally [37]. The consistent prominence of social media and the internet as information sources in Egypt, Saudi Arabia, and Turkey highlights the dominance of online resources in disseminating sustainability-related information, while also underscoring the utilization of formal educational settings and training programs [14, 19, 30, 32, 38].

The educational program yielded a substantial immediate improvement in ESD knowledge, indicating effective initial knowledge transfer and high engagement, a pattern observed in similar interventions with Saudi Arabian professionals [23]. However, a 6-month follow-up revealed a decline, illustrating the forgetting curve [39]. Despite this decline, significant retention over time was still noted for fundamental concepts, like water resources, climate change, carbon footprint, circular economy, green dentistry, and sustainable practices, suggesting substantial initial learning gains. Conversely, fundamental waste management principles have shown high baseline scores, indicating a strong pre-existing foundational knowledge that likely stems from the long-standing emphasis on these topics in standard dental education and infection control protocols. Consequently, this area showed less pronounced knowledge gains from the intervention, a finding consistent with studies in Jordan, Thailand, and India [26, 33, 40, 41]. This differential retention underscores the impact of prior knowledge and concept complexity on knowledge decay, emphasizing the need for reinforcement strategies to ensure sustained application [42].

Participants' ESD practices also significantly improved post-intervention, demonstrating the program's effectiveness in enhancing practical skills. Although a slight, non-significant decrease occurred at the 6-month follow-up, scores remained significantly higher than baseline, suggesting a degree of behavioral change overtime. This pattern of rapid initial adoption followed by gradual consolidation, aligns with skill acquisition trajectories, often characterized by a plateau or a slight decline as participants integrate new practices into their established routines, while facing real-world application challenges and difficulty in sustaining all new behaviors over time. This is consistent with findings in Peruvian dental professionals [42]. More importantly the long-term retention of most skills observed in this study may be attributed to the multi-component educational intervention, an approach supported by the literature as being more effective for lasting behavioral change and application [43].

An intermediate association suggests that as knowledge scores improved, practice scores also tended to be higher. This indicates that educational programs can effectively promote both cognitive understanding and practical skills. a finding supported by Hassan et al. [32]. However, this contrasts with studies reporting a disconnect between knowledge and implementation [19, 33, 34, 37] underscoring the complexity of translating awareness into action.

The regression analysis further identified higher educational level (5th level) and basic sustainability knowledge as significant predictors of good knowledge level, while simply hearing about sustainability and using social media/websites were associated with good practice, echoing findings from a Saudi Arabian study [38].

Hence, this study underscores significant initial gaps in KP of ESD among undergraduate students, while demonstrating the effectiveness of a targeted educational program in achieving substantial improvements in both domains. The observed knowledge decay and the knowledge-practice correlation highlight the necessity for integrating comprehensive sustainability education into undergraduate dental curricula, coupled with ongoing reinforcement and practical training to enhance lasting behavioral change and equip future dentists for environmentally responsible practice. The findings also emphasize the potential role of digital platforms in disseminating information and the need for regulatory frameworks to promote sustainable practices within the dental profession.

### Limitations and points of strength

This study has some limitations. First, the sample consisted of undergraduate dental students from a single institution, which may limit the generalizability of the findings to other student populations or practicing dentists. Second, the reliance on self-reported data may introduce recall bias or social desirability bias. Third, the study design lacked a control group to accurately measure the effect of the educational program. Finally, the students' prior knowledge of sustainability was fragmented. Although no dedicated courses exist, students likely acquired indirect information from other subjects, which may have influenced baseline scores. Future research should aim to better distinguish between these incidental and dedicated learning sources.

Despite these limitations, study possesses several notable strengths. First, it contributed to filling a critical gap in dental education regarding ESD by establishing a successful educational program. Second, the educational program demonstrated a significant impact on both knowledge and self-reported practices, highlighting its potential to foster positive behavioral change among future dentists. Third, the study of longitudinal design, with a six-month follow-up, allowed for the assessment of KP retention, providing valuable insights into the program effectiveness overtime. Moreover, the research developed the first comprehensive questionnaire assessing KP regarding ESD, based on the FDI guidelines, which was tested in a pilot feasibility and reliability study and had acceptable reliability.

## Conclusions

This study demonstrated the effectiveness of a targeted educational program in significantly improving dental students' initially limited KP of ESD. Correlation between knowledge and practice post-training reinforces the link between understanding and action. Students in clinical phase of education and those with prior awareness of sustainability were more likely to exhibit good baseline knowledge. Students who had information about sustainability, especially via social media/websites, exhibited a higher likelihood of good practice. Therefore, based on the effectiveness of our program, it is recommended that sustainability education be integrated into formal dental curricula and professional development activities.

## Supplementary Information

Supplementary Material 1. Study questionnaire.

Supplementary Material 2. Figures of correlation.

Supplementary Material 3. Tables showing KP scores according to participants’ educational level.

## Data Availability

The datasets used and/or analyzed during the current study are available from the corresponding author on reasonable request.

## Abbreviations

4Rs: Reduce, reuse, recycle, and rethink
ANOVA: Analysis of variance
ESD: Environmentally sustainable dentistry
FDI: World Dental Federation (French: *Fédération dentaire internationale*)
KAP: Knowledge, attitude and practice
KP: Knowledge and practice
KSA: Kingdom of Saudi Arabia
OHC: Oral health care
PUA: Pharos University in Alexandria
SDG: Sustainable development goals
SPSS: Statistical package for the social sciences
SWM: Solid waste management
UAE: United Arab Emirates
UK: United Kingdom
US: United States
