# Supplementary material for: Effectiveness of an educational program on improving the knowledge and practice of environmental sustainability in dentistry among undergraduate students at Faculty of Dentistry in Egypt: an interventional study
Source: BMC Med Educ. 2026 Jan 5;26:143. doi: 10.1186/s12909-025-08137-z (PMC12849658; doi:10.1186/s12909-025-08137-z)
Supplement: Supplementary file 1 — Supplementary Material 1. Study questionnaire. [file 12909_2025_8137_MOESM1_ESM.docx]

**Name: ____________________________________ ID_____________________**

Go Green for Sustainable Dentistry

**This questionnaire aims to assess knowledge and practice of undergraduate dental students in Pharos University regarding environmental sustainability in oral health care for research purposes. Your participation is voluntary, all data will be kept confidential.**

| **[I]** | **SOCIODEMOGRAPHIC and BASIC DATA** | | | | |
| --- | --- | --- | --- | --- | --- |
| **1** | **Age** |  | | | |
| **2** | **Educational Level** | 3^rd^ | 4^th^ | | 5^th^ |
| **3** | **Sex** | Male | | Female | |
| **4** | **Area of Residency** | Rural | | Urban | |
| **5** | **Have you participated in any events, workshops, or activities about “Sustainable Development” before?** | Yes | | No | |
| **6** | **How do you assess your knowledge about the concept of 'sustainability' in dentistry?**   1. I never heard about the concept of sustainability before this questionnaire. 2. I only heard about the concept of sustainability, but I have little information about it. 3. I have some knowledge about the basic concept of the sustainability approach. 4. I received training and have some experience in sustainability approach. 5. I have good experience with the sustainability approach that I can teach it to others. | | | | |
| **7** | **If you know about ‘sustainable dentistry’, please mention your source of information:**  a) University studies (curriculum) b) Awareness campaigns/ Trainings  c) Social media/ Websites d) Colleagues and relatives  e) From this questionnaire f) Other, (mention:____________________) | | | | |
| **[II]** | **KNOWLEDGE: “Choose one answer only”** | **Yes** | **No** | | **I don’t know** |
| **1** | **Solid waste can be reduced, reused, or recycled.** |  |  | |  |
| **2** | **Waste segregation (separation) doesn’t help in solid waste management.** |  |  | |  |
| **3** | **Improper disposal of solid waste can lead to pollution of rivers, lakes, and wells.** |  |  | |  |
| **4** | **The amount of water available for use around the world is limited.** |  |  | |  |
| **5** | **Climate change can occur due to burning fuel and consuming electricity.** |  |  | |  |
| **6** | **Energy depletion (exhaustion) has positive social and economic consequences.** |  |  | |  |
| **7** | **Climate change affects the oral health of populations** |  |  | |  |
| **8** | **Carbon footprint is a measure of the total amount of CO_2_ emissions caused by a system, event, or activity.** |  |  | |  |
| **9** | **How do you define ‘Sustainability’?**   1. **Focusing on short-term goals and early achievements.** 2. **Meeting current needs without harming future generations.** 3. **Depleting natural resources like raw materials.** 4. **I don't know.** | | | | |
| **10** | **What is meant by “Circular Economy”?**   1. Repeating the manufacturing process several times to obtain the best quality. 2. Keeping materials in use for longer lifespans through reuse, repair, and recycling. 3. An economic system based on resource extraction, production, consumption,& disposal. 4. I don’t know. | | | | |
| **11** | **Green dentistry is an approach that:**   1. Focuses solely on using natural ingredients in dental procedures. 2. Prioritizes cost-saving measures for dental practices. 3. Reduces the environmental impact of dental practices and involves a service model that supports and maintains wellness. 4. I don’t know | | | | |
| **12** | **Which of the following methods would be effective in reducing the dental industry's carbon footprint?**   1. Several appointments for low-risk patients. 2. Never combine visits of patients from the same family. 3. When buying dental supplies for the clinic, use combined instead of single shipments. 4. I don’t know. | | | | |
| **13** | **What characteristic makes the materials used in dentistry more environmentally friendly?**   1. Production using non-renewable resources. 2. Biodegradability and compostability. 3. High resistance to degradation. 4. I don’t know | | | | |
| **14** | **Which of the following is considered more sustainable in dentistry?**   1. Single-used instruments are more sustainable than reusable instruments. 2. Amalgam is more sustainable than glass ionomer materials. 3. Digital X-ray machines are more sustainable than conventional X-ray machines. 4. I don’t know | | | | |
| **15** | **Which dental waste could be hazardous to the environment?**   1. Bamboo toothbrushes, since they release nutrients into the soil when they decompose. 2. Alginate, since it is biocompatible and biodegradable. 3. Amalgam, since it contains 50% mercury and more than 20% silver. 4. I don’t know. | | | | |
| **16** | **The best method for managing radiographic X-ray film waste is:**   1. Autoclaving then disposing it in regular solid waste. 2. Sending it to incineration or landfill. 3. Recycling through local programs. 4. I don’t know. | | | | |
| **17** | **An appropriate method for managing amalgam waste is:**   1. Disposing it directly into the drain wastewater. 2. Reinstalling amalgam separator. 3. Disposing it in regular waste bin. 4. I don’t know. | | | | |
| **18** | **Intraoral scanners, CAD/CAM and 3D printing machines are all technologies that provide precise and durable dental restorations; looking at their environmental impact, they are:**   1. More eco-friendly than traditional methods. 2. Less eco-friendly than traditional methods. 3. Have the same environmental impact like traditional methods. 4. I don’t know | | | | |
| **III** | **Practice (Choose one answer only)** | **Always** | **Some-times** | | **Never** |
| **1** | **I use means of transport that run by fossil fuel (gasoline, diesel, liquified petroleum gas)** |  |  | |  |
| **2** | **I use active transportation (cycling, walking, …etc.)** |  |  | |  |
| **3** | **I switch off lights and electric devices when they are not in use** |  |  | |  |
| **4** | **I use renewable sources of energy (like solar energy)** |  |  | |  |
| **5** | **I use water faucets with aerators or with motion sensor to reduce water consumption** |  |  | |  |
| **6** | **I print on both sides of papers** |  |  | |  |
| **7** | **I reuse notebook papers or any previously used papers several times to reduce paper consumption** |  |  | |  |
| **8** | **When purchasing items I put into consideration their environmental impacts** |  |  | |  |
| **9** | **I choose manufacturers that follow an eco-friendly production strategy** |  |  | |  |
| **10** | **I buy products made from recycled materials** |  |  | |  |
| **11** | **I use disposable (single use) diagnostic sets instead of stainless steel autoclavable instruments** |  |  | |  |
| **12** | **I use disposable sterilization bags made from paper and plastic** |  |  | |  |
| **13** | **I use metallic air/water nozzles and suction tips that can be sterilized.** |  |  | |  |
| **14** | **I segregate (separate) waste into several bins at the clinic** |  |  | |  |
| **15** | **I dispose needles, endodontic files, and broken glass into a puncture proof container** |  |  | |  |
| **16** | **I dispose used X-ray film packet into the regular trash bin** |  |  | |  |
| **17** | **I buy pre-capsulated amalgam alloy** |  |  | |  |
| **18** | **I use rubber dam during insertion or removal of amalgam filling** |  |  | |  |
| **19** | **I dispose amalgam remnants into the regular trash** |  |  | |  |
| **20** | **I dispose amalgam remnants into the drain (wastewater)** |  |  | |  |
| **21** | **I dispose X-ray fixer solution down the drain (wastewater)** |  |  | |  |
| **22** | **I perform dental health education and prevention among several community groups**  Thank you |  |  | |  |
