# Supplementary material for: Effectiveness of an educational program on improving the knowledge and practice of environmental sustainability in dentistry among undergraduate students at Faculty of Dentistry in Egypt: an interventional study
Source: BMC Med Educ. 2026 Jan 5;26:143. doi: 10.1186/s12909-025-08137-z (PMC12849658; doi:10.1186/s12909-025-08137-z)
Supplement: Supplementary file 2 — Supplementary Material 2. Figures of correlation. [file 12909_2025_8137_MOESM2_ESM.docx]

**Table (8): Correlation between Knowledge vs. Practice in each period (n = 175)**

|  | **Pre** | | **Post** | | **3M** | |
| --- | --- | --- | --- | --- | --- | --- |
|  | **r** | **p** | **r** | **p** | **r** | **p** |
| **Knowledge vs. Practice** | 0.064 | 0.403 | 0.164^*^ | 0.030^*^ | 0.442^*^ | <0.001^*^ |

**r: Pearson coefficient**

*: Statistically significant at p ≤ 0.05

**Figure (1): Correlation between knowledge vs. practice in pre (n = 175)**

**Figure (2): Correlation between knowledge vs. practice in post (n = 175)**
