# Supplementary material for: Effectiveness of an educational program on improving the knowledge and practice of environmental sustainability in dentistry among undergraduate students at Faculty of Dentistry in Egypt: an interventional study
Source: BMC Med Educ. 2026 Jan 5;26:143. doi: 10.1186/s12909-025-08137-z (PMC12849658; doi:10.1186/s12909-025-08137-z)
Supplement: Supplementary file 3 — Supplementary Material 3. Tables showing KP scores according to participants’ educational level. [file 12909_2025_8137_MOESM3_ESM.docx]

**Table (1a): The relationship between level of knowledge and educational level
(n = 175) (Pre-intervention)**

|  | **Level of** **Knowledge** | | | | | | **χ^2^** | **^MC^ p** |
| --- | --- | --- | --- | --- | --- | --- | --- | --- |
|  | **Poor (n = 76)** | | **Moderate (n = 77)** | | **Good (n = 22)** | |  |  |
|  | **No.** | **%** | **No.** | **%** | **No.** | **%** |  |  |
| **Educational Level** |  |  |  |  |  |  |  |  |
| 3 | 68 | 89.5 | 59 | 76.6 | 15 | 68.2 | 6.890^*^ | 0.032^*^ |
| 5 | 8 | 10.5 | 18 | 23.4 | 7 | 31.8 |  |  |

χ^2^: **Chi square test MC: Monte Carlo test**

p: p-value for the comparison of knowledge across different educational levels

*: Statistically significant at p ≤ 0.05

**Table (1b): The relationship between total score for knowledge and educational level (n = 175) (Pre-intervention)**

|  | **No.** | **Total Score for** **Knowledge** | **t** | **p** |
| --- | --- | --- | --- | --- |
|  |  | **Mean ± SD.** |  |  |
| **Educational Level** |  |  |  |  |
| 3^rd^ | **142** | 10.56 ± 3.02 | 2.733^*^ | 0.007^*^ |
| 5^th^ | **33** | 12.21 ± 3.59 |  |  |

SD: **Standard deviation** **t: Student t-test**

p: p value for the comparison of knowledge across different educational levels

*: Statistically significant at p ≤ 0.05

**Table (2a): The relationship between level of practice and educational level
(n = 175) (Pre-intervention)**

|  | **Level of** **Practice** | | | | | | **χ^2^** | **^MC^p** |
| --- | --- | --- | --- | --- | --- | --- | --- | --- |
|  | **Good (n = 2)** | | **Fair (n = 116)** | | **Poor (n = 57)** | |  |  |
|  | **No.** | **%** | **No.** | **%** | **No.** | **%** |  |  |
| **Educational Level** |  |  |  |  |  |  |  |  |
| 3^rd^ | 44 | 77.2 | 96 | 82.8 | 2 | 100.0 | 1.025 | 0.610 |
| 5^th^ | 13 | 22.8 | 20 | 17.2 | 0 | 0.0 |  |  |

χ^2^: **Chi square test MC: Monte Carlo test**

p: p value for the comparison of practice across different educational levels

**Table (2b): The relationship between total score for practice and educational level (n = 175) (Pre-intervention)**

|  | **No.** | **Total Score for** **Practice** | **t** | **p** |
| --- | --- | --- | --- | --- |
|  |  | **Mean ± SD.** |  |  |
| **Educational Level** |  |  |  |  |
| 3^rd^ | **142** | 23.60 ± 4.48 | 2.024^*^ | 0.045^*^ |
| 5^th^ | **33** | 21.76 ± 5.60 |  |  |

SD: **Standard deviation** **t: Student t-test**

p: p value for the comparison of practice across different educational levels

*: Statistically significant at p ≤ 0.05
